# Navigating neurological re-emergence in feline infectious peritonitis: challenges and insights from GS-441524 and remdesivir treatment

**DOI:** 10.1177/20551169251360625

**Published:** 2025-09-19

**Authors:** Celia C de Witt Curtius, Maxime Rodary, Regina Hofmann-Lehmann, Andrea M Spiri, Marina L Meli, Aline Crespo Bouzon, Jennifer Wenk, Ilaria Cerchiaro, Benita Pineroli, Simon A Pot, Katrin Beckmann, Tatjana Chan, Manuela Wieser, Stefan Unterer, Sandra Felten, Solène M Meunier

**Affiliations:** 1 Clinical Laboratory, Department of Clinical Diagnostics and Services, and Center for Clinical Studies, Vetsuisse Faculty, University of Zurich, Zurich, Switzerland; 2 Clinic for Small Animal Medicine, Department of Emergency Medicine and Intensive Care, Vetsuisse Faculty, University of Zurich, Zurich, Switzerland; 3 Ophthalmology Section, Equine Department, Vetsuisse Faculty, University of Zurich, Zurich, Switzerland; 4 Clinic for Small Animal Medicine, Department of Neurology, Vetsuisse Faculty, University of Zurich, Zurich, Switzerland; 5 Clinic for Diagnostic Imaging, Department of Clinical Diagnostics and Services, Vetsuisse Faculty, University of Zurich, Zurich, Switzerland; 6 Department of Clinical Diagnostics and Services, Department of Anaesthesiology, Vetsuisse Faculty, University of Zurich, Zurich, Switzerland; 7 Clinic for Small Animal Internal Medicine, Vetsuisse Faculty, University of Zurich, Zurich, Switzerland

**Keywords:** Feline infectious peritonitis, feline coronavirus, meningoencephalomyelitis, relapse, GS-441524, remdesivir, alpha-1-acid glycoprotein, serum amyloid A

## Abstract

**Case summary:**

A 6-month-old male British Longhair cat presented with acute neurological signs, ocular changes, massive ascites and laboratory parameters consistent with feline infectious peritonitis (FIP). Systemic and neurological signs fully resolved with initial treatment (GS-441524; BOVA UK [15 mg/kg PO q24h for 42 days], levetiracetam [20 mg/kg q8h] and prednisolone [1 mg/kg q24h until day 21]). Lethargy and fever reappeared 17 days after treatment. Four days later, severe multifocal neurological signs re-emerged. High-field MRI revealed multifocal intra-axial and intramedullary lesions in the brainstem and cervical spinal cord, severe meningitis and generalised mild ventriculomegaly. Feline coronavirus (FCoV) RNA was detected in the cerebrospinal fluid by reverse transcription quantitative PCR (RT-qPCR). Abdominal effusion was absent. Serum alpha-1-acid glycoprotein (AGP) was again elevated. FIP re-emergence was suspected, and antiviral treatment was resumed. After 1 day of GS-441524 treatment (15 mg/kg PO q24h), severe hypoventilation developed, requiring intubation and mechanical ventilation for 1.5 days. Treatment was switched to remdesivir (16.7 mg/kg IV q24h, Veklury; Gilead) for 4 days. Oral GS-441524 was then reintroduced (10 mg/kg q12h) and continued until day 84. Treatment resulted in partial recovery with moderate ataxia and reduced left-sided menace response remaining 181 days after starting the second treatment.

**Relevance and novel information:**

This case illustrates the complexity of diagnosing and treating re-emerging FIP-associated neurological signs. AGP monitoring offers a promising non-invasive approach for early detection of relapse. By adapting short- and long-term antiviral treatment and providing intensive care, excellent long-term outcomes can be obtained for cats with severe relapsing FIP-related neurological signs.

## Introduction

Although new effective oral antiviral drugs often lead to excellent short-term recovery and long-term remission in cats with feline infectious peritonitis (FIP), the recurrence of clinical signs is possible.^1–5^ Cats with FIP-related neurological manifestations are considered harder to treat and have a higher risk of recurrence because of the presumed limited drug penetration across the blood‒brain barrier;^6,7^ therefore, careful monitoring during and after antiviral treatment is crucial. MRI and cerebrospinal fluid (CSF) analysis have proven useful but are associated with limited availability, high costs and the need for general anaesthesia.^6^ Non-invasive monitoring of serum alpha-1-acid glycoprotein (AGP) and serum amyloid A (SAA) levels could benefit cats with FIP-related neurological manifestations, as well as those with cavitary effusions.^8^ Evidence is lacking regarding the optimal treatment for recurrent FIP-associated neurological signs. We describe the follow-up and successful treatment of a cat with re-emergent FIP-associated neurological signs despite initial successful oral antiviral treatment with GS-441524.

## Case description

### Signalment and history

**Figure 1:**
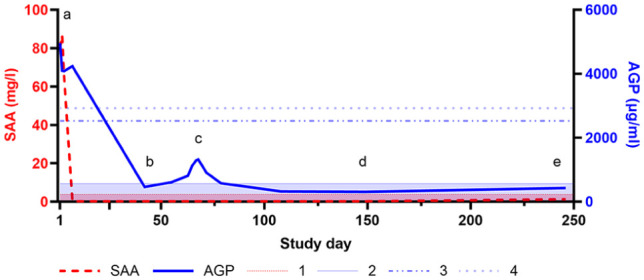
Time course of serum amyloid A (SAA) (thick dashed line, on the left side) and serum alpha-1-acid glycoprotein (AGP) (thick solid line, on the right side) concentrations. 1 = reference interval (RI) SAA: <3.9 mg/l; 2 = RI AGP: <567 µg/ml; 3 = AGP in the range of 2531–2927 µg/ml supports suspicion of feline infectious peritonitis (FIP) in a cat with compatible clinical signs (sensitivity 61%, specificity 80%); 4 = AGP >2927 µg/ml strongly supports FIP in a cat with compatible clinical signs (sensitivity 54%, specificity 97%).^8^ (a) Initial presentation (day 1); (b) end of first treatment (day 42); (c) re-emergent FIP signs (day 63); (d) end of second treatment (day 149/84; days since initiation of the first/second treatment, respectively); (e) last recheck (day 246/181)

A 6-month-old intact male British Longhair cat, kept indoors with a partner, initially presented with acute-onset neurological signs, ocular changes and abdominal distension. On admission, the cat showed lethargy, ventroflexion, severe bilateral uveitis, active chorioretinitis, mild ataxia and a single self-limiting generalised tonic‒clonic seizure (Table 1). Complete blood count (CBC) and serum chemistry, including elevated AGP (4966 µg/ml) and SAA (80.6 mg/l), suggested FIP (Table 2, Figure 1). Abdominal ultrasound revealed generalised lymphadenomegaly and massive corpuscular ascites. Abdominocentesis yielded protein-rich transudate with a positive Rivalta test and AGP of 3650 µg/ml. High posi-tive feline coronavirus (FCoV) reverse transcription quantitative PCR (RT-qPCR) confirmed FIP, with viral loads of 3.0 × 10^8^ copies/ml in ascites and 5.9 × 10^5^ copies/ml in blood.^9^ Central nervous system (CNS) imaging and CSF analysis were not performed. Oral antiviral treatment was initiated (15 mg/kg q24h for 42 days, GS-441524; BOVA UK), with prior approval by the governmental veterinary office (TVB number ZH124/2022; 34964). Supportive care included fluids, maropitant, ondansetron, mirtazapine, prednisolone (1 mg/kg q24h until day 21 and then tapered), long-term levetiracetam (20 mg/kg q8h) and ophthalmological treatment of the anterior uveitis with topical prednisolone (until day 21, paused on days 4–7 because of corneal erosions), tropicamide (until day 42) and lubricating eye drops. Colistin, and specifically cidofovir (a DNA virus-targeting cytidine analogue), were added for suspected feline herpesvirus-1 keratitis on day 21. Full systemic and neurological resolution was obtained, although inactive chorioretinal and iris-lens lesions persisted (Table 1). By day 42, all blood parameters had normalised and FCoV RT-qPCR from blood was negative for viral RNA, supporting the decision to discontinue treatment (Figure 1).

**Table 1:** Clinical and neurological signs at initial presentation (day 1), at the end of the first treatment (day 42) and at the time of diagnosis of feline infectious peritonitis (FIP) re-emergence (day 66/1)

Parameter	Day 1 (initial presentation)	Day 42 (end of first treatment)	Day 66/1 (re-emergent FIP)
Body condition score	2/9	3/9	4/9
Mental status	Lethargic, responsive	Alert, responsive	Obtunded
Posture	Ventroflexion	Normal posture	Intermittent left-sided head tilt
Gait	Slight ataxia	Normal gait	Non-ambulatory tetraparesis
Proprioception/ hopping	Prompt (all four limbs)	Prompt (all four limbs)	Reduced (all four limbs)
Cranial nerves	Multiple deficits	Normal	Absent menace response, oculocephalic and nasocortical reaction
Seizures	Tonic–clonic seizure	No seizures	No seizures
Ascites	Severe	Mild (not procurable)	None
Ocular status	Uveitis, chorioretinitis	Healed uveitis, inactive chorioretinitis	Not performed

**Table 2:** Comparison of blood work at the initial presentation (day 1 of first treatment), at the time of re-emergence of feline infectious peritonitis (FIP)-associated clinical signs (day 63) and at the last follow-up (day 246/181 since initiation of the first/second treatment, respectively)

Parameter	Initial presentation (day 1)	Presentation as a result of re-emergent FIP signs (day 63)	Last control visit (day 246/181)	RI*
Hct (%)	**20**	44	**29**	33–45
Hgb (g/dl)	**6.5**	13.7	**10.8**	11.3–15.5
RBC (×10^6^/µl)	**5.08**	**11.06**	8.42	7.0–10.7
MCHC (g/dl)	33	**31**	37	33.0–36.0
MCV (fl)	**38**	40	**35**	40.0–48.0
WBC (×103/µl)	**5.7**	8.1	**13.1**	4.6–12.8
Reticulocytes (/µl)	3556	n/a	5052	–
PLT (×10^3^/µl)	**29 (no aggregates)**	413	**177 (many aggregates)**	180.0–680.0
Band neutrophils (×10^3^/µl)	**0.97**	n/a	n/a	0.0–0.1
Segmented neutrophils (×10^3^/µl)	4.61	n/a	n/a	2.32–10.01
Lymphocytes (×10^3^/µl)	**0.14**	2.31	3.39	1.05–6.0
Total bilirubin (µmol/l)	**45.6**	<2.5	<2.5	0.1–3.5
Glucose (mmol/l)	6.4	**1.9** ^†^	4.9	4.0–9.0
Urea (mmol/l)	**7.3**	**6.7**	10.8	7.4–12.6
Creatinine (µmol/l)	**37**	**97**	142	98.0–163.0
Total protein (g/l)	**62**	69	67	64.0–80.0
Albumin (g/l)	**22**	34	34	32.0–42.0
Globulins (calculated) (g/l)	39	35	33	–
A/G	0.56	0.97	1.03	–
Cholesterol (mmol/l)	4.7	2.9	3.1	2.6–6.8
Triglycerides (mmol/l)	**2.8**	0.5	0.4	0.3–1.3
ALP (U/l)	28	36	40	16.0–43.0
DGGR-lipase (U/l)	8	8	13	6.0–21.0
AST (U/l)	**124**	26	**17**	19.0–44.0
ALT (U/l)	51	**27**	**26**	34.0–98.0
CK (U/l)	324	109	112	77.0–355.0
Sodium indirect (mmol/l)	155	152	153	150.0–157.0
Potassium (mmol/l)	**3.6**	5.3	4.1	3.8–5.4
Chloride indirect (mmol/l)	120	**111**	117	113.0–123.0
Calcium (mmol/l)	**2.21**	2.48	2.52	2.4–2.8
Phosphate (mmol/l)	**2.05**	**2.69**	**1.91**	0.9–1.8
SAA (mg/l)	**80.6**	<0.3	1.3	<3.9
AGP (µg/ml)	**4966**	**810**	428	<567^‡^
FCoV RT-qPCR blood (copies/ml)	**5.9 x 10^5^**	0	0	–
FCoV RT-qPCR feces (copies/swab)	**4.3 x 10^4^**	0^§^	**9.6 x 10^5^**	–
Anti-FCoV antibody titre (measured by IFA)^¶^	**1/400**	**1/400**	**1/1600**	–

Bold numbers indicate values outside the reference interval (RI) or positive results in RT-qPCR or IFA

* RIs apply to adult cats

† Blood sample was 2 days old (sent by referring veterinarian) – glucose may be unreliable

‡ If FIP is suspected (because of a compatible history, clinical signs and laboratory changes), alpha-1-acid glycoprotein (AGP) >2927 µg/ml strongly supports FIP, AGP in the range of 2531–2927 µg/ml supports suspicion and AGP <2531 µg/ml makes FIP less likely but does not exclude it (cutoffs: 2927 µg/ml: sensitivity 54%, specificity 97%; 2531 µg/ml: sensitivity 61%, specificity 80%)^8^

§ Results from day 65 owing to insufficient sample material on day 63

¶ <1/25 = negative

A/G = albumin/globulin ratio; ALP = alkaline phosphatase; ALT = alanine aminotransferase; AST = aspartate aminotransferase; CK = creatine kinase; DGGR = 1,2-O-dilauryl-rac-glycero glutaric acid-(6′-methylresorufin) ester; FCoV = feline coronavirus; Hct = haematocrit; Hgb = haemoglobin; IFA = immunoflurescence assay; MCHC = mean cell haemoglobin concentration; MCV = mean cell volume; n/a = information not available; PLT = platelet count; RBC = red blood cell count; RI = reference interval; RT-qPCR = reverse-transcription quantitative PCR; SAA = serum amyloid A; WBC = white blood cell count

### Re-emergent FIP signs

At 17 days after treatment (day 59), lethargy and elevated temperature were reported. The cat was presented 21 days after treatment (day 63) to the referring veter-inarian, with rapidly progressing ataxia. Neurological examination on day 66 at our hospital revealed severe deficits consistent with multifocal neuroanatomical localisation including the brainstem (Table 1). Owing to poor clinical conditions, no ophthalmologic examination was performed.

### Diagnostic imaging and laboratory findings at re-emergent FIP

**Figure 2:**
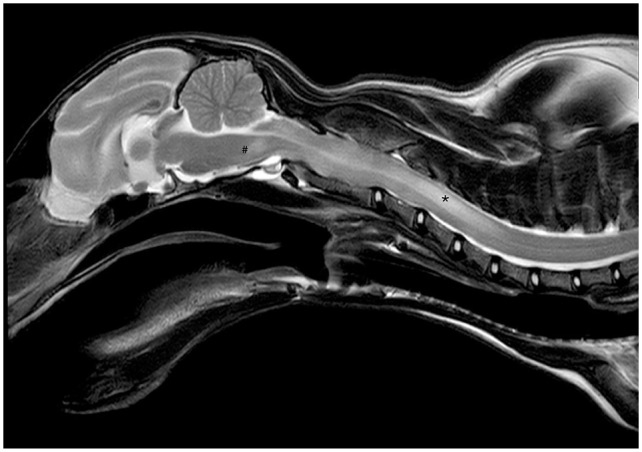
Sagittal T2-weighted (T2W) image of the brain and cervical spine. There was moderate spinal cord swelling and an extensive, asymmetrical, T2W and T1-weighted hyperintense lesion in the mesencephalon (#) extending along the cervical spinal cord until C5. The lesion is most pronounced at the level of C4 (*)

**Figure 3:**
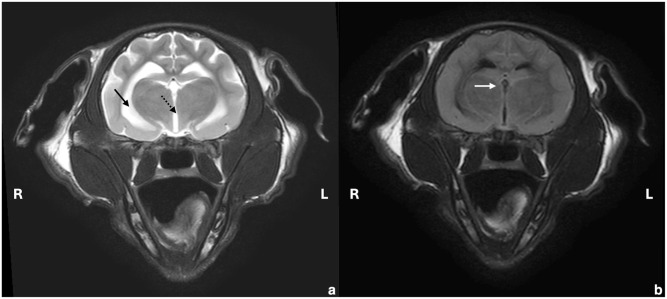
Transverse (a) T2-weighted and (b) T2-fluid attenuation inversion recovery (FLAIR) images of the brain at the level of the thalamus and third ventricle. The ventricular system is moderately widened (arrow: lateral ventricle, dashed arrow: third ventricle) with incomplete cerebrospinal fluid suppression in the FLAIR sequence (white arrow)

**Figure 4:**
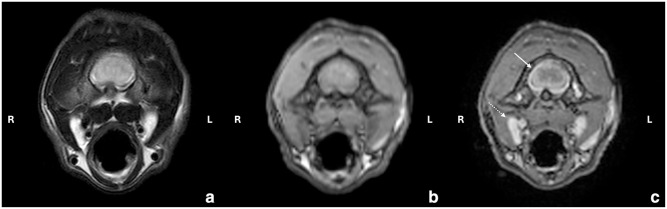
Transverse (a) T2-weighted, (b) T1W precontrast and (c) T1-weighted (T1W) postcontrast images of the cervical spinal cord at the level of C1. The meninges are moderately thickened with strong contrast enhancement (white arrow), which is compatible with marked meningitis. Mild bilateral medial retropharyngeal lymphadenomegaly (dashed white arrow)

**Figure 5:**
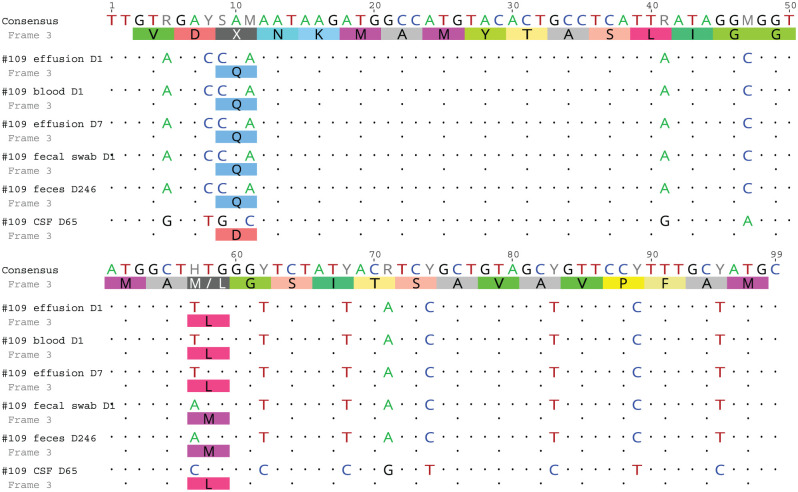
Nucleotide (top; consensus) and translated amino acid sequences (bottom; frame 3) of the analysed feline coronavirus strains. Dots depict the same nucleotide and amino acid sequences, whereas letters represent differences with respect to the consensus sequence. The sequences were edited and aligned via Clustal Omega,^12^ and images were created with Geneious Prime 2020.2.5

On day 63, blood analysis revealed only elevated AGP (810 µg/ml) (Table 2), with no effusion present. High-field MRI of the brain and cervical spine was performed on day 66/1 (days since initiation of the first/second treatment, respectively; going forward, time points will be presented in this format). Intramedullary lesions extending from the medulla oblongata to C5, spinal cord swelling (Figure 2), moderate ventriculomegaly (Figure 3) and multifocal thickening of the meninges with strong contrast enhancement (Figure 4) were found. The swelling restricted access to the occipital cistern, necessitating a lumbar CSF puncture instead of an atlanto-occipital approach. CSF analysis revealed moderate lymphocytic pleocytosis (341 nucleated cells/µl) and severely elevated total protein levels (18.7 g/l). No infectious organisms or atypical cells were observed. FCoV RT-qPCR was lowly positive (2.5 × 10^3^ copies/ml), strongly suggesting FIP relapse with meningoencephalomyelitis. Viral sequen-cing of the spike gene (99 bp) revealed the M1058L mutation associated with systemic spread and/or FIP^10,11^ in effusion, blood (day 1) and CSF (day 66/1) but not in faeces (day 1 and day 246/181) (Figure 5). In addition to the M1058L mutation, early blood, effusion and faecal sequences (day 1) matched the late faecal sample (day 246/181) at the nucleotide level. In contrast, the CSF sequence (day 66/1) differed by 14/99 nucleotides from the effusion and blood sequences (day 1), with 13 syn-onymous and one non-synonymous mutation. Thus, the CSF sequence (day 66/1) differed from the blood and effusion sequences (day 1) by only 1/32 amino acids.

### Treatment challenges of re-emergent FIP

Antiviral treatment was resumed (GS-441524, 15 mg/kg PO q24h) and mannitol was given once for suspected intracranial hypertension. On day 67/2, the cat’s mentation worsened, accompanied by bradypnea (<16 breaths/min), shallow breathing, bradycardia (106 beats/min) and hypothermia (35.1°C). Blood pressure remained normal. Owing to an absent swallowing reflex and severe hypercapnia (venous partial pressure of CO_2_ [pCO_2_] of 134.3 mmHg) secondary to hypoventilation, the cat was anaesthetised, intubated and placed on positive pressure-controlled mechanical ventilation with a fraction of inspired oxygen (FiO_2_) of 30%. Total intravenous anaesthesia (TIVA) combined with propofol, dexmedetomidine and midazolam ensured deep sedation. Oral GS-441524 was replaced with intravenous remdesivir (16.7 mg/kg q24h, Veklury; Gilead). After 22 h, respiratory drive improved, allowing TIVA discontinuation and spontaneous breathing. Serial venous blood gas examinations at 4, 8 and 12 h after extubation revealed pCO_2_ levels of 64.8, 79.3 and 76.8 mmHg, respectively. To prevent hypercapnia-associated hypoxemia, oxygen was supplied via an oxygen box (FiO_2_ 50%). The cat was tetraparetic with reduced mentation but had a normal respiratory rate and improved breathing. By day 69/4, the neurological status improved, allowing the transition to oral GS-441524 at an increased dose (10 mg/kg q12h). Oxygen supplementation was stopped when the haemoglobin saturation reached 100% under room air. Supportive medications included prednisolone (1 mg/kg IV q12h), levetiracetam, vitamin B1, ondansetron (until day 71/6), maropitant (until day 76/11) and mirtazapine (until day 67/2). Amoxicillin-clavulanic acid was initiated from day 67/2 until day 73/8 for suspected aspiration pneumonia.

### Long-term follow-up

By day 77/12, the cat could stand and walk, although severe generalised ataxia persisted. The cat was discharged on day 79/14, but manual bladder expression remained necessary. Spontaneous micturition was first reported on day 83/18. Prednisolone was tapered and discontinued on day 100/35. By day 107/42, blood results were within reference intervals, and FCoV RT-qPCR from blood was negative. Owing to persistent neurological deficits, such as severe ataxia, slightly reduced tactile placing on the right side, a reduced menace response on the left side and a mildly reduced oculocephalic reflex (see video 1 in the supplementary material), antiviral treatment was extended to 84 days. By day 149/84, owners reported the cat jumping onto windowsills and daily improvements in mobility and coordination (see videos 2 and 3 in the supplementary material). The remaining reduced left-sided menace response, wide-based stance and moderate ataxia were attributed to residual CNS damage (alternative injury) and no longer associated with acute FIP. The second GS-441524 treatment was stopped. FCoV RT-qPCR from faeces was weakly positive (day 149/84), contrasting expectation of complete FCoV absence after antiviral treatment.^13^ On day 246/181, only mild vestibular ataxia remained. Blood work was unremarkable, with mild deviations of questionable clinical relevance and a negative FCoV RT-qPCR result (Table 2).

## Discussion

### Relapse vs reinfection

When clinical signs of FIP re-emerge, distinguishing between relapse and reinfection may influence the therapeutic approach. Relapse can be defined as recurrence due to incomplete viral clearance, possibly involving viral resistance. It occurs shortly after treatment, involves the same virus strain and may require higher dosages or longer treatment. Conversely, reinfection refers to a cat acquiring a new infection with a different or the same FCoV strain.^14^ Prior FCoV infection does not confer protective immunity, allowing reinfection despite high antibody levels.^15,16^ A reinfected cat may still respond to the initial treatment. In our case, although only a small portion of the FCoV virome was sequenced, the results suggest the presence of a similar virus in the CSF at D-66/1 (only one amino acid change in addition to synonymous mutations). The rapid recurrence of clinical signs and the absence of outdoor access or exposure to another FCoV support relapse. Identical faecal viral sequences from day 1 and day 246/181 indicate re-emergence but do not rule out reinfection by the same FCoV strain circulating in the household, as the partner cat may have been shedding FCoV but was not tested.

### Risk factors and rate of re-emerging FIP

**Figure 6:**
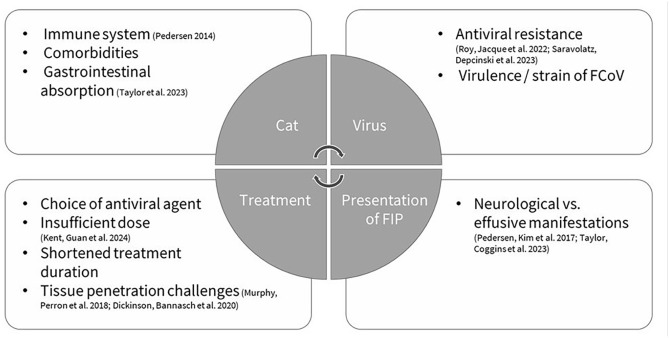
Known and suspected risk factors for re-emergence of feline infectious peritonitis (FIP).^1,5–7,16,18–20^ FCoV = feline coronavirus

The incidence of re-emergent clinical signs during and after FIP treatment varies widely in the literature, with a range of 0–73.7% (Table 3).^1–5,17^ No comparison has been made between cats with and without initial FIP-related neurological manifestations. Known and suspected risk factors for FIP re-emergence can be grouped into cat-, virus-, clinical presentation- and treatment-related factors (Figure 6). These findings, along with varying follow-up lengths, probably explain the differences in the results of these studies. Among drug-related factors, the choice of antiviral agent may be relevant (Figure 6). In this case, lower GS-441524 concentrations in the CSF are thought to result from limited blood‒brain barrier penetration, which has led to the empirical recommendation of higher dosages for FIP-related neurological manifestations.^6,7,21^ A recent study using an unlicensed GS-441524-like treatment reported promising results: 42.3% of cats with neurological or ocular FIP-related manifestations respond-ed positively, and only 4.7% died.^2^

**Table 3:** Reported rates of feline infectious peritonitis (FIP) re-emergence

Study	FIP re-emergence rate (cats with FIP-related neurological manifestations at re-emergence of clinical signs)	Study includes neurological cases	Total number of cats (cats with FIP-related neurological manifestations at study inclusion, if applicable)	Treatment
Ongoing bi-centre study (UZH and LMU)*	1% (1/2, 50%)	Yes	202^†^ (40/202, 19.8%)	GS-441524 and remdesivir^‡^
Buchta et al., 2024*	2.5% (0/1, 0%)	No	40 (n/a)	GS-441524^‡^
Taylor et al, 2023^5^	10.8% (17/33, 51%)	Yes	307 (62/306, 20%)	Remdesivir and GS-441524^†^
Coggins et al, 2023^4^	16% (n/a)	No	28 (n/a)	Remdesivir and GS-441524^†^
Jones et al, 2021^2^	12.7% (n/a)	Yes	393 (103/393, 26%)	GS-441524 (unlicensed)
Krentz et al, 2021^17^	0% (n/a)	No	18	GS-441524 (Xraphconn)
Yin et al, 2021^3^	3.3% (n/a)	Yes	30 (n/a)	GS-441524 and GC 376
Pedersen et al, 2017^1^	73.7% (n/a)	No	19	GC 376

Data in parentheses are n (%)

* Unpublished data

† Status as of October 2024

‡ BOVA UK

LMU = Ludwig Maximilian University Munich; n/a = information not available; UZH = University of Zurich

### AGP: an early non-invasive monitoring biomarker

AGP is a moderate acute-phase protein that plays a critical role in the inflammatory response, with peak levels occurring approximately 2–3 days after activation.^22^ It has shown promise for monitoring treatment response and predicting FIP re-emergence.^8^ Retrospective analysis of AGP levels, in this case, revealed a gradual increase before FIP re-emergence, although values remained below the previously defined FIP-suggestive cutoff. Moreover, all other blood parameters remaining within reference intervals or deviations were considered not clinically relevant, highlighting the potential of AGP as an early, non-invasive monitoring parameter.

### Optimal treatment protocol for FIP-related neurological manifestations

The optimal treatment dose and duration for cats with FIP-related neurological manifestations still need to be determined. The initial treatment protocol in this case followed a recent study showing excellent outcomes in cats with FIP-related cavitary effusions.^23^ Rapid clinical improvement and normalisation of all laboratory parameters during treatment support its initial treatment efficacy. However, the recurrence of FIP-related clinical signs 17 days after the end of treatment raises the question of whether a longer treatment duration, higher dose or increased dosing frequency might have led to more sustained remission. FCoV RT-qPCR monitoring in the CSF before discontinuing the first treatment could have revealed incomplete viral clearance,^6^ but was not performed because of its invasiveness regarding normalisation of neurological status.

## Conclusions

This case highlights the complexity of diagnosing and treating re-emerging FIP-related neurological signs. Regular non-invasive rechecks, including AGP monitoring, allow early detection. Short-term intravenous remdesivir treatment associated with intensive care, followed by a prolonged 84-day course of high-dose oral GS-441524, led to excellent long-term outcomes despite the initially critical situation.
